# Studying Collagen Architecture in Solution by Raman Optical Activity Spectroscopy

**DOI:** 10.1021/acs.analchem.5c07017

**Published:** 2026-03-02

**Authors:** Jiří Kessler, Jaroslav Šebestík, Martin Šafařík, Radek Pelc, Petr Bouř, Tao Wu

**Affiliations:** 89220 Institute of Organic Chemistry and Biochemistry, Czech Academy of Sciences, Flemingovo náměstí 2, Prague 16 000, Czech Republic

## Abstract

Raman and Raman optical activity (ROA) spectroscopy provide a unique insight into the three-dimensional structure of biomacromolecules; however, it is often hampered by low sensitivity, low resolution, and the lack of theoretical models. To advance the methodology, we demonstrate that it can discriminate between two collagen proteins, types I and II. The data are interpreted on the basis of molecular modeling correlated with spectra of five synthetic collagen-type peptides serving as simple models. In the peptides, accurate density functional theory (DFT) calculations and correlation of the structure with the spectra are possible, allowing us to determine convenient marker bands linking spectral intensities to the molecular architecture. ROA spectra reflect the polyproline II (PPII) helical conformation of the peptide’s main chain and indicate subtle concentration-dependent structural variations in type I collagen. Several vibrational bands originating from proline (Pro), hydroxyproline (Hyp), and the Pro–Hyp–Gly motifs can be related to the collagen triple helix core. ROA spectroscopy thus captures several aspects of collagen’s chirality, enables the study of solvent effects and dynamics, and is expected to aid connective tissue studies.

## Graphical abstract

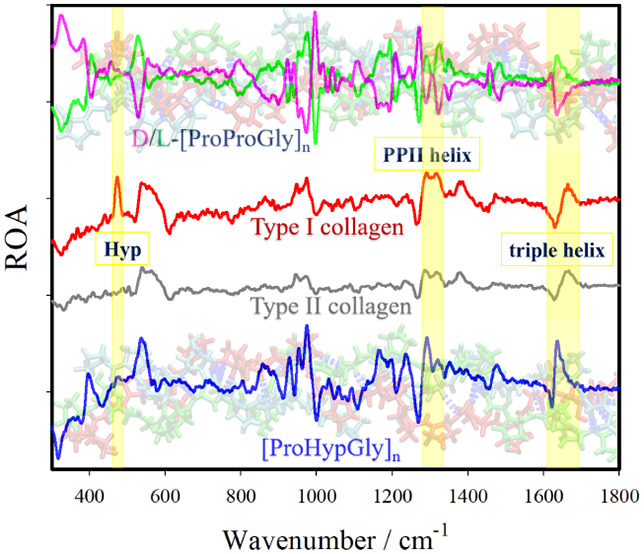

## Introduction

Collagen is the most abundant protein in the animal kingdom and is predominantly found in connective tissue, such as tendons, skin, cartilage, and bone. Its fine structure varies; fibril-forming collagen types I, II, III, V, and XI are the most important forms involved in aging and in many pathophysiological processes such as cancer and cystic fibrosis. Collagen fibers also play a key role in biomineralization by providing the scaffold for bone and dentin formation. Their highly ordered packing and hierarchical bundling lend collagen a remarkable mechanical resilience *in vivo* and even explain its long-term durability in archaeological findings.^1^

A key structural feature of collagen is the triple helix, in which three polypeptide strands adopt polyproline II-type (PPII) geometry. Proline (Pro, P), hydroxyproline (Hyp, H), and glycine (Gly, G) are its most common amino acids, and the ProHypGly (PHG) triplet is instrumental in assembling the helical chains.^2^ Despite decades of research, many aspects of collagen fibrillization remain incompletely understood.^3^ Even the most advanced protein-structure prediction algorithms such as AlphaFold2 ^4^ and AlphaFold3 ^5^ struggle to model higher-order collagen helices and fibrils, thus highlighting the need for more fundamental approaches that probe self-assembly across scales.^6^

The structure and physicochemical properties of collagen helices have been extensively studied.^7^ The triple-helical nature of collagen was established by diffraction studies in the 1950s.^8,9^ However, elucidation of its structure at atomic resolution appeared difficult, and an alternative approach employing collagen-type peptides as simple models was introduced in the late 1960s.^10^ This led to the verification of the intramolecular hydrogen bonds between N–H­(Gly) and O=C­(Pro), stabilizing the triple helix using X-ray diffraction of collagen-type peptide crystals.^11^ Numerous crystallography studies of collagen-type peptides were conducted thereafter.^12–15^

Besides X-ray crystallography, other techniques used to study collagen proteins comprise scanning electron microscopy (SEM),^16^ atomic force microscopy (AFM),^17^ mass spectrometry (MS),^18^ and infrared (IR)^19,20^ and Raman^21^ spectroscopy. Unlike IR, Raman spectroscopy is very suitable for studying aqueous solutions. It is also label-free and nondestructive. As such, it has been widely used not only to characterize collagen in terms of its secondary structure but also to study the extracellular matrix, of which collagen is the main component.^21^

An even better structural sensitivity is achieved by Raman optical activity (ROA) spectroscopy, which measures a tiny intensity difference between scattered right- (*I*_R_) and left- (*I*_L_) circularly polarized light. It has been developed as an efficient analytical approach to study organic molecules and biomolecules^22^ as well as metal complexes.^23^ Weak signal is ROA spectroscopy’s inherent limitation. For example, the ROA/Raman ratio [a so-called normalized circular intensity difference, CID = (*I*_R_ – *I*_L_)/(*I*_R_ + *I*_L_)] is typically below 1 × 10^–3^. Yet modern spectrometers do make it possible to detect the ROA signal even in samples of limited solubility, such as collagen. Another constraint is related to interpretation of the spectra, almost exclusively relying on *ab initio* modeling. Even here, however, the density functional theory (DFT), Cartesian tensor transfer, or similar fragment-based methods make the computations applicable to large molecules including biopolymers.

Previously, X-ray diffraction was used to distinguish between different collagen types,^24^ often in conjunction with IR and Raman spectroscopy.^25–28^ However, these techniques are blind to molecular chirality, quite insensitive to minor conformational differences (IR, Raman) and “contraindicated” in solutions (X-ray diffraction). In this context, ROA spectroscopy is often a convenient complementary technique.

In the present study, we thus employ ROA spectroscopy jointly with molecular modeling to elucidate the structure of collagen proteins (types I and II) and five newly synthesized collagen-type peptides. Being highly sensitive to backbone conformation, the technique provides rich insight into the secondary and tertiary structure of these complex biomolecules in an aqueous milieu. The combination of experimental ROA spectra with theoretical modeling makes it possible to reliably assign characteristic spectral features to specific conformational motifs.

## Experimental Section

Collagen type I from rat tail tendon and collagen type II from chicken sternal cartilage were purchased as powders from Sigma-Aldrich and dissolved in 0.1 M acetic acid. Although collagens I and II are both fibril-forming, collagen II has better solubility in 0.1 M acetic acid at room temperature. Three different concentrations (13, 23, and 50 mg/mL) were prepared for type I. The 50 mg/mL concentration could be used as well but required warming the sample to 50 °C, which led to a change in the native conformation.

### Peptide Synthesis

The synthesis of the five collagen-type peptides was performed using the Fmoc/tBu protocol (Fmoc = 9-Fluorenylmethoxycarbonyl)^29^ on an automatic solid-phase synthesizer ABI 433A (Applied Biosystems) using the FastMoc 0.1 mmol program (SynthAssist version 3.1) with a single coupling: 10 equiv. of an excess of protected amino acids, the *o*-(Benzotriazol-1-yl)-*N*,*N*,*N′*,*N′*-tetramethyluronium hexafluorophosphate coupling reagent, and 20 equiv. of an excess of diisopropylethylamine were used. H-Ala-HMPB-ChemMatrix and H-Gly-HMPB-ChemMatrix resins were used, with a loading of ca. 0.4 mmol/g. Hydroxyproline (Hyp) was incorporated by using Fmoc-Hyp­(tBu)-OH protection. The cleavage of the peptides from the resin was carried out by a mixture of trifluoroacetic acid (4.5 mL), H_2_O (150 mL), 1,2-ethanedithiol (150 mL), thioanisole (150 mL), and triisopropyl silane (50 mL) for 4 h. Peptide deprotection was carried out simultaneously. All of the peptides were obtained at >90% purity.

### Chromatographic and MS Methods

Molecular weight of peptide fragments was determined using matrix-assisted laser desorption ionization time-of-flight mass spectrometry (MALDI-TOF-MS). An instrument with a quaternary pump, thermostat, diode array detector, and reverse-phase C_18_ columns was used for HPLC analysis. Purification of the peptides was carried out by semipreparative HPLC on the VYDAC 250 × 10 mm, 10 μm RP-18 column at a flow rate of 3 mL/min using a 0–100% ACN (acetonitrile) gradient in 0.05% aqueous TFA. HPLC analysis was performed on a Poroshell 120 SB-C18 2.7 μm, 3.0 × 50 mm column at a flow rate of 1 mL/min and diode array detection using a 5-5-100-100% gradient of ACN in 0.05% aqueous TFA within 0-1-11-13 min. The injection peak was not integrated (Figure S1).

(1) PXGA-triacontapeptide, (PHG)_9_PHA—H-(Pro-Hyp-Gly)_9_-Pro-Hyp-Ala-OH—HPLC: R_T_ 3.2 min. For C_121_H_174_N_30_O_41_ (2703.25), found MALDI-MS *m*/*z*: 2704.2 ([M + H]^+^) (Figure S2).
(2) PPGA-triacontapeptide, (PPG)_9_PPA—H-(Pro-Pro-Gly)_9_-Pro-Pro-Ala-OH—HPLC: R_T_ 4.0 min. For C_121_H_174_N_30_O_31_ (2543.30), found MALDI-MS *m*/*z*: 2544.1 ([M + H]^+^) (Figure S3).
(3) ppG-triacontapeptide, D-(PPG)_10_—H-(d-Pro-d-Pro-Gly)_10_–OH—HPLC: R_T_ 4.0 min. For C_120_H_172_N_30_O_31_ (2529.28), found MALDI-MS *m*/*z*: 2531.1 ([M + 2]^+^) (Figure S4).
(4) XPGA-triacontapeptide, (HPG)_9_HPA—H-(Hyp-Pro-Gly)_9_-Hyp-Pro-Ala-OH—HPLC: R_T_ 3.1 min. For C_121_H_174_N_30_O_41_ (2703.25), found MALDI-MS *m*/*z*: 2704.4 ([M + H]^+^) (Figure S5).
(5) GXPA-hentriacontapeptide, (GHP)_10_A—H-(Gly-Hyp-Pro)_10_-Ala-OH—HPLC: R_T_ 3.1 min. For C_123_H_177_N_31_O_42_ (2760.27), found MALDI-MS *m*/*z*: 2761.3 ([M + H]^+^) (Figure S6).

### Raman/ROA Spectroscopy

Raman and backscattered circular polarization (SCP) ROA spectra were acquired at room temperature on a BioTools ROA spectrometer (laser excitation: 532 nm, resolution: 7 cm^–1^) in a cuvette of ∼120 μL volume made of fused silica. The laser power was within the range of 200–1000 mW, and accumulation times were 8 to 72 h. To quench the fluorescence, all samples were irradiated at 200 mW for several hours before measurements, Raman intensities normalized to the water peak at 1650 cm^–1^, and the remaining fluorescence background was corrected by polynomial baseline.

## Theoretical Calculations

Initial geometry of the (PHG)_9_PHA and (PPG)_9_PPA peptides was extracted from the crystal structure of collagen-like peptides.^11,13^ Optimization and subsequent calculation of partial atomic charges by the Merz–Kollman method were carried out at the DFT level using the B3PW91 functional with the 6-31++G** basis set, CPCM water solvent model, and empirical GD3 dispersion, using the Gaussian 16 program.^30^ The partial atomic charges were refined by the RESP procedure^31^ using the Antechamber program from the Amber 18 package.^32^ Each peptide was placed in a periodic box (120 × 50 × 50 Å) filled with water molecules using the Packmol program.^33^

The systems were minimized, equilibrated, and then the final 10 ns molecular dynamics (MD) run was carried out using the GAFF force field,^34^ periodic boundary conditions, a canonical ensemble (NVT), a temperature of 300 K, and a 1 fs time step.

The entire collagen-type peptide was cut into a set of partially overlapping fragments containing 4 or 7 amide units and offset by one amide. For example, the first 4-peptide fragment included amides 1 to 4, the second one included amides 2 to 5, etc.^35^ The fragments were partially optimized using the normal mode coordinates,^36^ and Raman and ROA tensors and force fields were calculated at the B3PW91/6-31++G**/CPCM­(water) level. Then, the tensors were transferred back to the original molecule and to another 1000 snapshots to account for the MD dispersion of the geometry. Backscattered Raman and ROA intensities were calculated at the harmonic level and averaged. The intensities were convoluted with Lorentzian peaks (full width at half maximum, FWHM 10 cm^–1^) while taking into account the Boltzmann temperature factor at 300 K.

## Results and Discussion

Experimentally, ROA spectra of collagen required extended acquisition times due to their inherently weak signals. Although laser light may theoretically affect the sample, no laser-induced degradation was detected in the Raman spectra. However, they exhibit a concentration dependence. Raman spectra of collagen I (23 mg/mL) and collagen II (25 mg/mL) are almost identical except for the low wavenumber (200–500 cm^–1^) bands (Figure 1).

**Figure 1:**
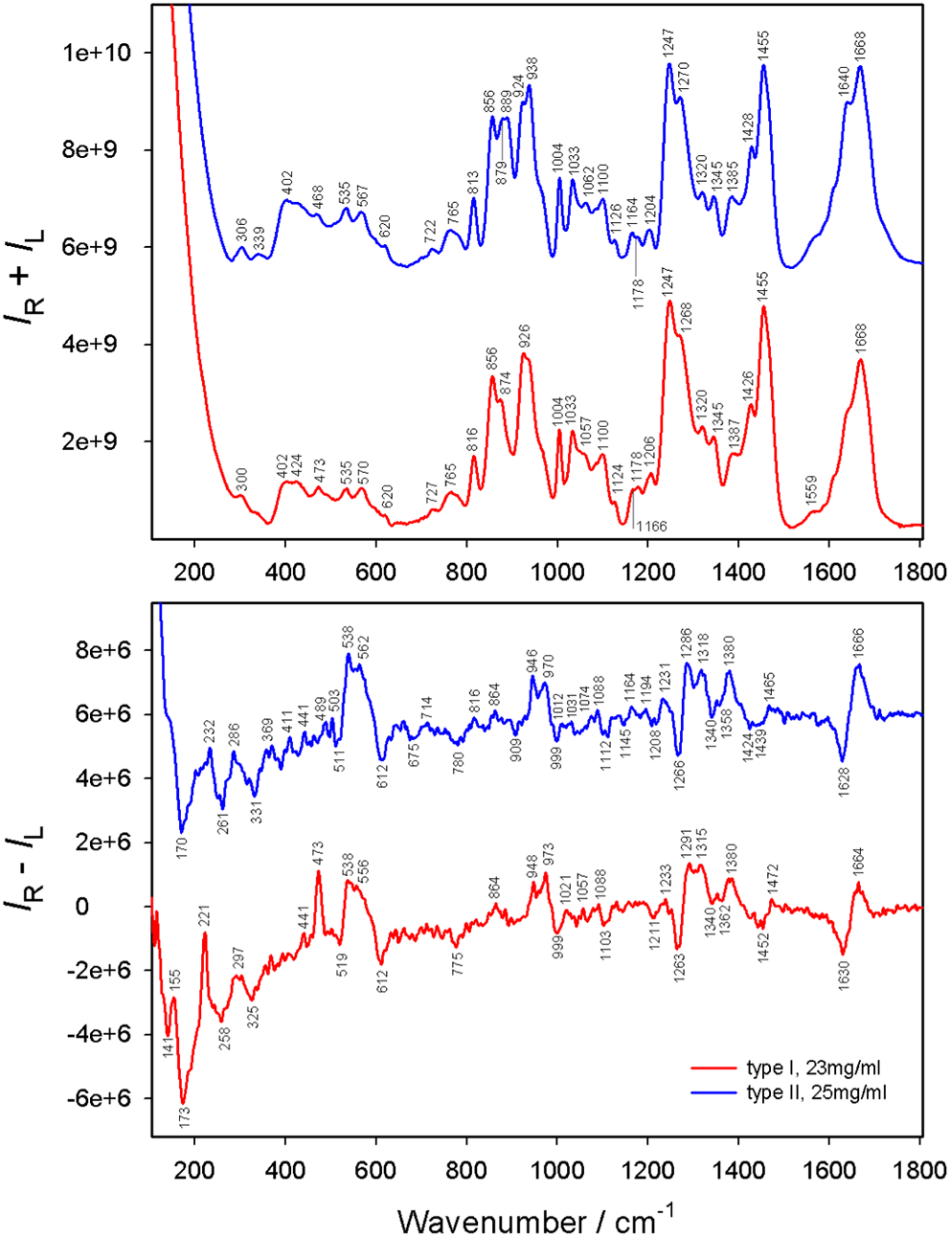
Raman (*I*_R_ + *I*_L_) and ROA (*I*_R_ – *I*_L_) spectra of collagen I (23 mg/mL) and collagen II (25 mg/mL) measured in 0.1 M acetic acid.

Raman spectrum of collagen I solution was slightly affected by local concentration variations due to viscosity. At a low concentration (13 mg/mL) of type I, there are no obvious Raman bands below 400 cm^–1^ (Figure 2). As the concentration increases to 23 mg/mL, the solution becomes more viscous, and a weak band appears at 300 cm^–1^, most likely due to the delocalized chain deformation, possibly coupled with vibrations of the aqueous solvent.^37^ A previous study has shown that irreversible denaturation occurs at the melting point of collagen (40 °C).^38^ At 50 °C, the solubility of collagen I can be as high as 50 mg/mL, this being the highest concentration of collagen I we used. Upon cooling such a high-concentration solution to room temperature (at which the measurements were carried out), a gel was formed in the spectroscopic cuvette. The weak band at 300 cm^–1^ becomes hardly visible, and a new band appears at 221 cm^–1^ (corresponding to delocalized peptide chain deformation^37,39^ ); it was not detected at 23 or 13 mg/mL. These spectral variations indicate that the structure of collagen I undergoes irreversible changes at a high temperature. Two small bands at 306 and 339 cm^–1^ are encountered in type II only and thus represent a spectral fingerprint (type II vs I). Major Raman and ROA bands of collagen proteins are given in Table 1.

**Figure 2:**
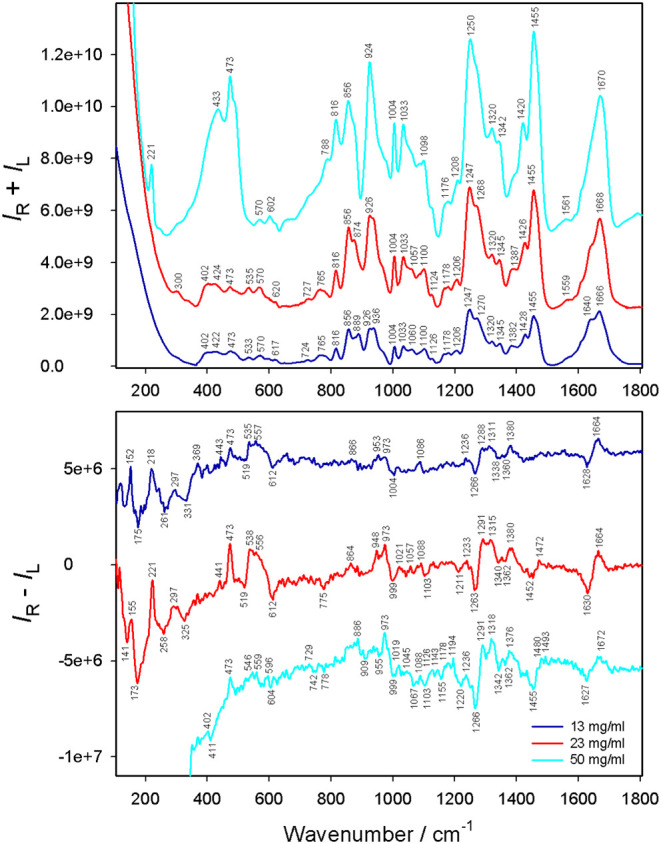
Raman (*I*_R_ + *I*_L_) and ROA (*I*_R_ – *I*_L_) spectra of collagen I (13, 23, 50 mg/mL) in 0.1 M acetic acid. Close-to-highest possible concentrations were used, and 50 mg/mL was obtained by briefly heating the sample beyond the denaturation temperature.

**Table 1:** Summary of the Major Raman and ROA Bands Observed for Collagen Types I and II in 0.1 M Acetic Acid

Wavenumber/cm^–1^				
Raman		ROA		
Type I^a^	Type II^b^	Type I^a^	Type II^b^	Band assignment^22,40^
1668	1668	1664 (+)	1666 (+)	Amide I
	1640	1630 (−)	1628 (−)	ν(C=O)
1455	1455	1472 (+)	1465 (+)	δCH_3_, δCH_2_
1426	1428	1452 (−)	1439 (−)	
1345	1345	1340 (−)	1340 (−)	C_α_–H, CH_2_ wagging
1320	1320	1315 (+)	1318 (+)	
1268	1270	1291 (+)	1286 (+)	Amide III, δN–H
1247	1247	1263 (−)	1266 (−)	
1033	1033	1021 (+)	1031 (+)	Phe; Hyp/Pro ring
		999 (−)	1012 (+)	
			999 (−)	
		973 (+)	970 (+)	Hyp/Pro ring deformation
926	938			ν(C–C) of protein backbone
	924			ν(C–C) of Pro ring
874	889	864 (+)	864 (+)	ν(C–C) of Hyp/Pro ring
856	856			
765	765	775 (−)	780 (−)	Amide IV
570	567	556 (+)	562 (+)	S-S vibrations; Hyp/Pro ring deformation
535	535	538 (+)	538 (+)	
473	468	473 (+)		Hyp ring deformation
300	339	325 (−)	331 (−)	Delocalized chain deformation
	306			

a 23 mg/mL.

b 25 mg/mL.

For collagen I, concentration-dependent Raman features include amide I vibrations: at 50 mg/mL a broad band appeared at 1670 cm^–1^, which was shifted to 1668 cm^–1^ at 23 mg/mL, and further split into two bands at 1666 and 1640 cm^–1^ at 13 mg/mL (Figure 2). Similar variations were observed for the amide III vibrations. While the gel-like sample (50 mg/mL) exhibited only a single band at 1250 cm^–1^, there were two peaks at the two lower concentrations (e.g., 1270, 1247 cm^–1^ at 13 mg/mL and 1268, 1247 cm^–1^ at 23 mg/mL).

The Raman intensity of the 473 cm^–1^ band (assigned to the Pro ring by other authors^21,41^ ) is much stronger at 50 mg/mL compared to 23 or 13 mg/mL. A weak Raman band was also discernible in collagen II at 468 cm^–1^. Although the Raman intensity is strongest in the gel-state sample (50 mg/mL), the corresponding ROA intensity is the weakest, i.e., the CID value is the smallest, suggesting the triple helical structure was strongly affected by the high temperature (50 °C) required to dissolve the sample (i.e., above the denaturation temperature of 40 °C, as discussed above).

ROA spectral patterns of collagen I (13 and 23 mg/mL) and collagen II (25 mg/mL) were quite similar to each other, except in the low wavenumber region (100–500 cm^–1^). The vibration modes of collagen I in this region are difficult to assign. ROA spectral shapes also slightly vary with concentration. Nevertheless, an intense band was recorded at 473 cm^–1^ in type I at all three concentrations (Figure 2). As it is missing in the collagen II spectrum (Figure 1), it can serve as an ROA fingerprint of collagen I.

Figure 3 shows the Raman and ROA spectra of the collagen-type peptides (PHG)_9_PHA, (PPG)_9_PPA, and D-(PPG)_10_, along with the calculated spectra for the first two. Raman and ROA spectra for all five synthesized collagen-type peptides are presented in Figure S7. Comparison of Raman and ROA bands for collagen proteins and the five collagen-type peptides is provided in Table S1 and Table S2, respectively. Apparently, the Raman spectra of the peptides and proteins (collagens) are more alike than the ROA spectra. The ROA spectrum of the (PHG)_9_PHA peptide is most similar to those of both collagen proteins (I or II). This includes couplets at ∼1610–1670 cm^–1^, ∼1260–1295 cm^–1^, ∼1080–1100 cm^–1^, and ∼970–1000 cm^–1^; positive bands at ∼1310–1320 cm^–1^, ∼945–955 cm^–1^, and 538 cm^–1^; and a negative band at ∼310–340 cm^–1^.

**Figure 3:**
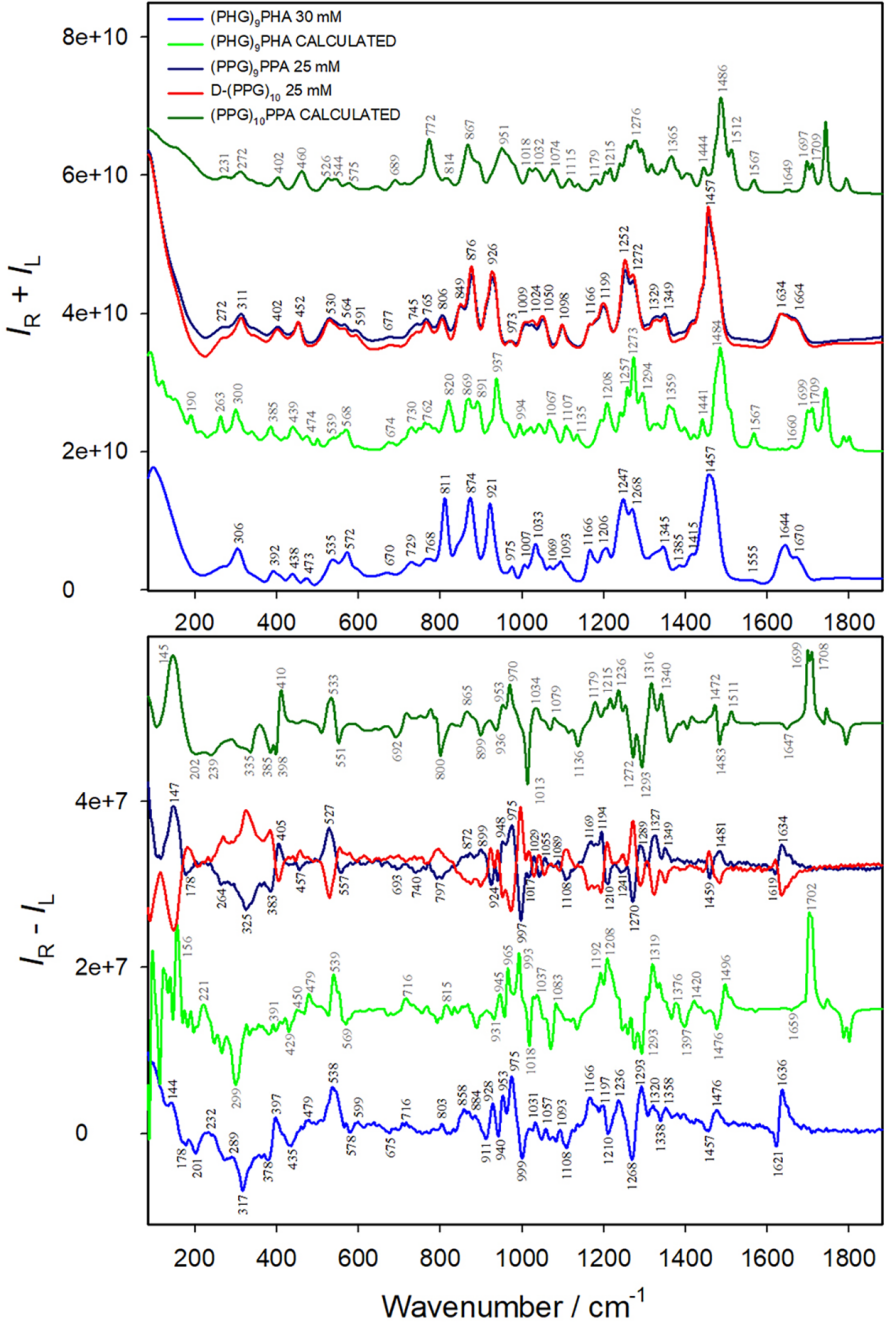
Raman (*I*_R_ + *I*_L_) and ROA (*I*_R_ – *I*_L_) spectra of three collagen-type peptides measured in 0.1 M acetic acid and calculated spectra of two of them.

ROA spectra of the (PPG)_9_PPA and D-(PPG)_10_ peptides are almost perfect ″mirror images” despite a minor composition difference (l-Ala instead of Gly). The amide I vibration mode of (PPG)_9_PPA exhibits a strong/weak positive/negative band at high/low wavenumber (1634/1619 cm^–1^). This ROA couplet is more easily discernible when compared with D-(PPG)_10_ than when measured alone and is well reproduced in the simulated spectrum (Figure 3). A similar couplet is often encountered in α-helical proteins.^42^ The amide I ROA bands of (PPG)_9_PPA strongly depend on the concentration. A very slight decrease from 25 to 20 mg/mL results in the ROA couplet (+/−) of amide I vibration being transformed into a positive-only band; the rest of the ROA bands remain mostly unaffected (Figure S8). The ROA spectral pattern is similar to the PPII form of polyproline,^37^ indicating that at low concentrations (PPG)_9_PPA preferably adopts a PPII conformation. This is consistent with the crystal structures of similar collagen-type peptides reported elsewhere.^11,13^

Among the five collagen-type peptides, the prominent Raman band found in collagen I at 473 cm^–1^ (Figure 2) was detected only in (PHG)_9_PHA (Figure 3 and Figure S7). It was noticeably weaker in the other two Hyp-containing peptides, (HPG)_9_HPA and (GHP)_10_A at 476 cm^–1^ and 479 cm^–1^, respectively. In the Hyp-free peptides, i.e., (PPG)_9_PPA and D-(PPG)_10_, it was absent altogether (as in other polyproline peptides^37^), and 452 cm^–1^ was its nearest equivalent. We may thus conclude^21,41^ that it is the Hyp rather than the Pro ring in the Hyp-Pro-Gly fragment that contributes to the above-mentioned 473 cm^–1^ band.

Raman bands at 1033 and 1004 cm^–1^ are typically assigned to phenylalanine (Phe).^43^ We have nevertheless detected both of them in collagen I and II (Figure 1), similarly to other authors.^44^ We also recorded very similar Raman bands at 1033 cm^–1^ and 1007 cm^–1^ in one of the Hyp-containing peptides (PHG)_9_PHA, but not in the Hyp-free peptides (PPG)_9_PPA and D-(PPG)_10_ or even the Hyp-containing peptides (HPG)_9_HPA and (GHP)_10_A, in which the amino acid sequence in the Pro-Hyp-Gly domain has been modified. This indicates that the Hyp residue may contribute to the 1033/1004 cm^–1^ bands of collagen proteins.

A Raman peak at 954 cm^–1^, previously assigned to Pro and used to differentiate between collagen types,^28^ was not discernible in collagen proteins and peptides measured in the present study. However, ROA bands could be clearly detected at ∼940–980 cm^–1^. This indicates that even collagen structures of the same type may differ depending on their source.

A strong Raman band at 623 cm^–1^ was recorded in the (HPG)_9_HPA peptide only (Figure S7). Incidentally, the peptide has a slightly lower solubility in acetic acid than (PHG)_9_PHA, (PPG)_9_PPA, and D-(PPG)_10_. Many ROA spectral features of collagen were also absent in the (HPG)_9_HPA and (GHP)_10_A peptides, such as the amide I couplet (1610–1670 cm^–1^) found in collagen I and II (Figure 1) and the (PHG)_9_PHA peptide (Figure 3). This may be due to the low concentration similar to (PPG)_9_PPA (20 versus 25 mM) (Figure S8), but another ROA couplet observed in the collagen proteins within the region of 1080–1100 cm^–1^ is also missing. In addition, a ROA negative band at 481 cm^–1^ was observed in (HPG)_9_HPA and 479 cm^–1^ in (GHP)_10_A, as opposed to the positive pattern at 473 cm^–1^ in collagen I and 479 cm^–1^ in (PHG)_9_PHA. These spectral patterns make the presence of Hyp-Pro-Gly and Gly-Hyp-Pro domains in the collagen structure rather unlikely. The Pro-Hyp-Gly and Pro-Pro-Gly domains are the most important components in collagen proteins, the former one being predominant.

Previous reports^44,45^ have attributed the Raman band at 535 cm^–1^ to S–S vibrations. In the denatured form of collagen I (Figure 2), many of the otherwise present Raman/ROA bands were not detected, most likely due to the effect of heating on the S–S bond. However, the Raman band at 535 cm^–1^ and a corresponding ROA band at 538 cm^–1^ in the (PHG)_9_PHA peptide (Figure 3) are almost identical to those encountered in collagen I and II proteins, and similar Raman and ROA bands were also recorded in the other four peptides (Figure S7). This indicates^37^ that the ν­(αC–N) vibration mode of the Hyp/Pro ring deformation strongly contributes to the Raman band at 535 cm^–1^.

Three parallel polypeptide strands in the left-handed PPII helical conformation form a right-handed triple collagen superhelix. One may expect to detect specific spectral patterns of both the PPII helix and the superhelix. Indeed, some studies have already characterized the PPII structure, and a strong positive ROA band in the range of 1314–1325 cm^–1^ has been detected.^22,46^ Positive ROA bands at 1315 cm^–1^ (intact collagen I at 23 mg/mL) and 1318 cm^–1^ (denatured collagen I at 50 mg/mL) are mostly due to C_α_–H and CH_2_ wagging (Figure 2). We recorded a similar band at 1318 cm^–1^ for collagen II (Figure 1), at 1320 cm^–1^ for the (PHG)_9_PHA peptide, at 1327 cm^–1^ for the (PPG)_9_PPA peptide, and at 1324 cm^–1^ for (HPG)_9_HPA and (GHP)_10_A peptides (Figure S7).

MD-simulated geometries of (PHG)_9_PHA and (PPG)_9_PPA in a water box (Figure S9) are close to the crystal structure of other collagen-type peptides.^11,13^ The histograms of segment lengths in Pro-Hyp-Gly in (PHG)_9_PHA and Pro-Pro-Gly in (PPG)_9_PPA are comparable, as revealed by MD simulations (Figure S10). Differences between the Ramachandran plots of Gly-Pro in the (PHG)_9_PHA peptide (Figure 4A) and (PPG)_9_PPA peptide (Figure 4D) are minimal. A replacement of Hyp for Pro in (PHG)_9_PHA translates to changes in the Ramachandran plot: Pro-Hyp (Figure 4B) versus Pro-Pro (Figure 4E) and Hyp-Gly (Figure 4C) versus Pro-Gly (Figure 4F). The left-handed PPII helical conformation of the three parallel strands in the two polypeptides is quite stable, and indeed, most of the observed ROA spectral patterns correspond to the PPII structure.

**Figure 4:**
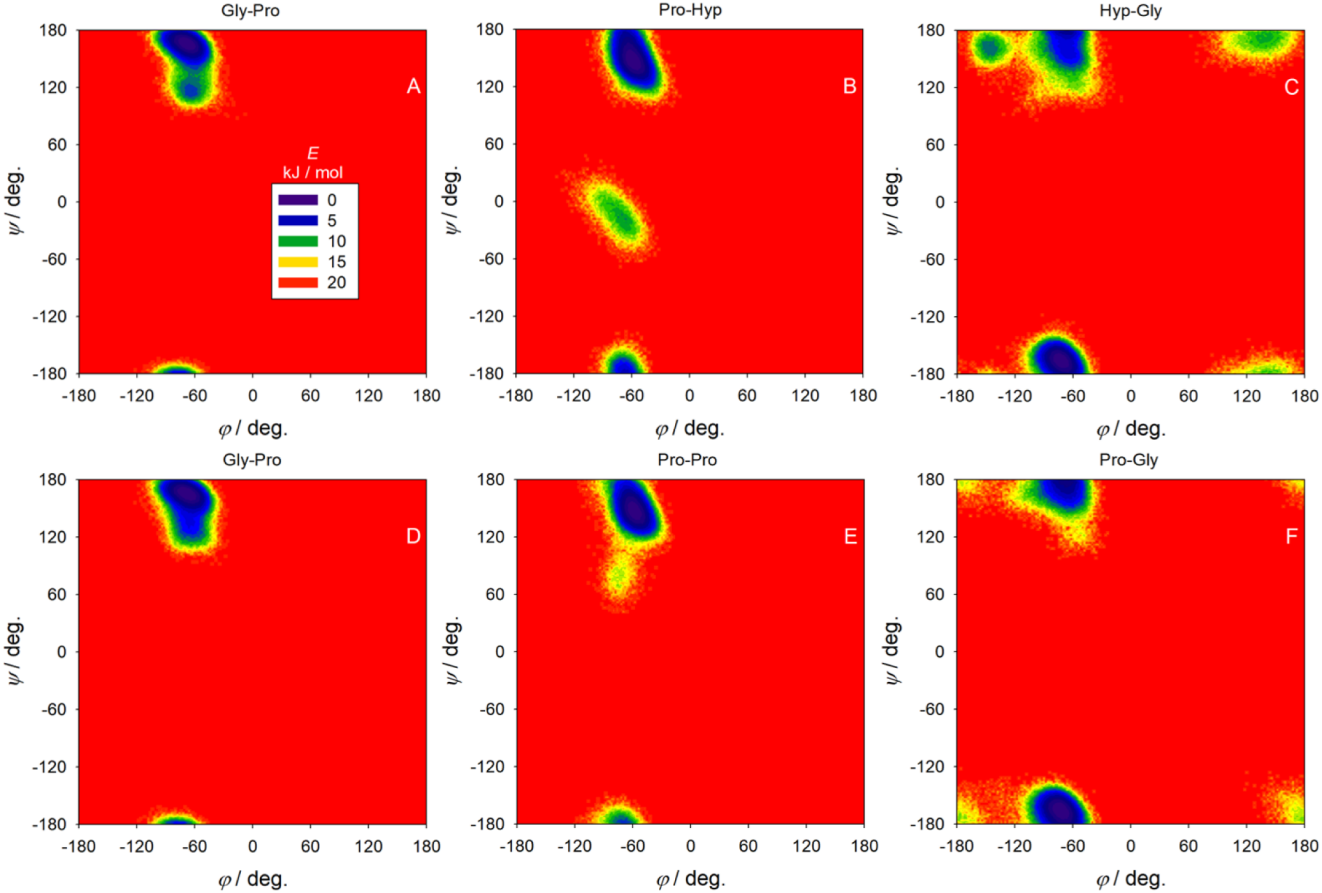
Dependence of the free energy on the (φ, ψ) angles, as obtained by MD in the Gly-Pro-Hyp-Gly sequence (A, B, C) in the (PHG)_9_PHA peptide, and the Gly-Pro-Pro-Gly sequence (D, E, F) in the (PPG)_9_PPA peptide.

Major ROA spectral patterns of collagen proteins and peptides are summarized in Table S2.^22,40^ The most important one is the ROA couplet of amide I vibrations (centered at ∼1650 cm^–1^, with a negative/positive part at low/high wavenumber). The amide I couplet, arising mainly from the C=O stretching mode specific to the triple collagen superhelix, is also summarized separately in Table S3. In particular, we compared the CID value at the high and low wavenumber components (CID_1_ vs CID_2_). The greatest CID value (∼9 × 10^–4^) was recorded in the (PHG)_9_PHA peptide at high wavenumber (1636 cm^–1^) and smallest (∼9 × 10^–5^) in the denatured collagen I also at high wavenumber (1672 cm^–1^). The difference of the two CID values (Δ_CID_ = CID_1_ – CID_2_) followed a similar pattern, with the peptide (PHG)_9_PHA featuring the highest (∼1.68 × 10^–3^) and denatured collagen I the smallest (∼3.5 × 10^–4^) values. The concentration dependence of the Δ_CID_ value for collagen I itself is prominent; the low/medium (13/23 mg/mL) concentrations yield the highest/moderate Δ_CID_. This indicates that the low concentration (less viscous state) favors stabilization of the triple helical structure. The Δ_CID_ value of the (PHG)_9_PHA peptide is slightly higher compared to either (PPG)_9_PPA or D-(PPG)_10_, indicating that the presence of Hyp contributes more strongly than Pro to the formation and stability of helical conformations.

Another ROA couplet (−/+) centered at ∼1100 cm^–1^ is also frequently attributed to the α-helix structure.^40^ We observed it as inverted (+/−) in collagens I and II (Figure 1), and the (PHG)_9_PHA and (PPG)_9_PPA peptides (Figure 3); it is hardly visible in (HPG)_9_HPA and (GHP)_10_A (Figure S7). Other spectral signatures previously observed^40,46^ for the α-helices included positive ROA bands at ∼1340–1345 cm^–1^, ∼1297–1312 cm^–1^, and ∼870–950 cm^–1^. While we did not record any positive ROA band at ∼1340–1345 cm^–1^ and ∼1297–1312 cm^–1^ (either for the collagen proteins or the peptides), we noticed a positive ROA band at 948 cm^–1^ in collagen I (at 23 mg/mL). In collagen II, we recorded a similar positive band at 946 cm^–1^, plus a negative band at 909 cm^–1^ (Figure 1). All five collagen-type peptides also exhibited both positive and negative ROA bands in this region (Figure S7). These spectral signatures are also seen in α-helices; thus, they cannot be used to identify collagen proteins and peptides.^40^

## Conclusions

We measured Raman and Raman optical activity (ROA) spectra of natural collagen as well as model peptides to study their structure in solution and used a multiscale computational protocol to interpret the experimental data. The spectroscopy confirmed that both type I and type II collagen predominantly adopt the polyproline II (PPII) helical conformation and that a characteristic amide I signature is indicative of the triple helical arrangement. These features are also found in the spectra of synthesized collagen-type peptides. Molecular dynamics (MD) and density functional theory (DFT) calculations provided a reasonable agreement between computed and experimental frequencies and intensities, thus constituting a reliable basis to interpret the experimental patterns in the peptide models and native collagen proteins. This combined experimental-and-theoretical approach makes it easier to identify spectral peaks in Raman and ROA spectra of collagen proteins and assign them to structural motifs. This is of particular relevance as no collagen crystals have been grown yet.

Raman and ROA spectra sensitively reflect the vibrational modes associated with proline (Pro) and hydroxyproline (Hyp) and confirm their cooperative role in stabilizing the Pro–Hyp–Gly motif within the collagen triple helix. For example, the Hyp-derived ROA marker band at 473 cm^–1^ makes it possible to distinguish type I from type II collagen, thus complementing earlier studies, in which this Raman band was empirically assigned to proline. The chiral Hyp fingerprint in the ROA spectra was confirmed by molecular modeling.

In the future, improvements in instrument sensitivity and molecular modeling accuracy are expected to further refine the assignment of spectral features to details of molecular architecture, dynamics, and interactions with the environment. The results obtained so far document the potential of Raman and ROA spectroscopy, when combined with molecular modeling, to relate molecular chirality to peptide folding and, to some extent, connective tissue architecture.

## Supplementary Material



## Notes

HPLC chromatograms of synthetic peptides, their MALDI, Raman, and ROA spectra, MD-simulated structure of two peptides, MD-simulated distribution of selected peptide segment distances, and a list of major Raman and ROA bands of collagen proteins and peptides (PDF)

## Abbreviations

ACN: acetonitrile
CID: circular intensity difference
DIPEA: diisopropylethylamine
DMF: *N*,*N*-dimethylmethanamide
EDT: 1,2-ethanedithiol
Fmoc: 9-fluorenylmethoxycarbonyl
HBTU: *o*-(benzotriazol-1-yl)-*N*,*N*,*N*′,*N*′-tetramethyluronium hexafluorophosphate
HOBt: *N*-hydroxybenzotriazole
HPLC: high-performance liquid chromatography
MALDI: matrix-assisted laser desorption/ionization
MS: mass spectrometry
ROA: Raman optical activity
tBu: *tert*-butyl
TFA: trifluoroacetic acid
TOF: time of flight (detection)
TIS: triisopropyl silane
VCD: vibrational circular dichroism
