# Supplementary material for: Studying Collagen Architecture in Solution by Raman Optical Activity Spectroscopy
Source: Anal Chem. 2026 Mar 2;98(10):7481–8. doi: 10.1021/acs.analchem.5c07017 (PMC13000884; doi:10.1021/acs.analchem.5c07017)
Supplement: Supplementary file 1 [file ac5c07017_si_001.pdf]

Supporting Information

Studying Collagen Architecture in Solution by Raman Optical Activity Spectroscopy

Jiří Kessler, Jaroslav Šebestík, Martin Šafařík, Radek Pelc, Petr Bouř, and Tao Wu*

Institute of Organic Chemistry and Biochemistry, Czech Academy of Sciences, Flemingovo náměstí 2, Prague 16 000, Czech Republic

**tao.wu@uochb.cas.cz*

Contents:

Figure S1. HPLC chromatograms of prepared peptides

Figure S2-S6. MALDI spectrum of peptide

Figure S7. Raman and ROA spectra of the five collagen-type peptides

Figure S8. Raman and ROA spectra of peptide $(PPG)_9PPA$ at different concentrations

Figure S9. MD simulated structure of peptides

Figure S10. MD simulated distribution of peptide segment distances

Table S1. Summary of major Raman bands for collagen proteins and peptides

Table S2. Summary of major ROA bands for collagen proteins and peptides

Table S3. ROA couplet attributed to amide I in collagen proteins and peptides

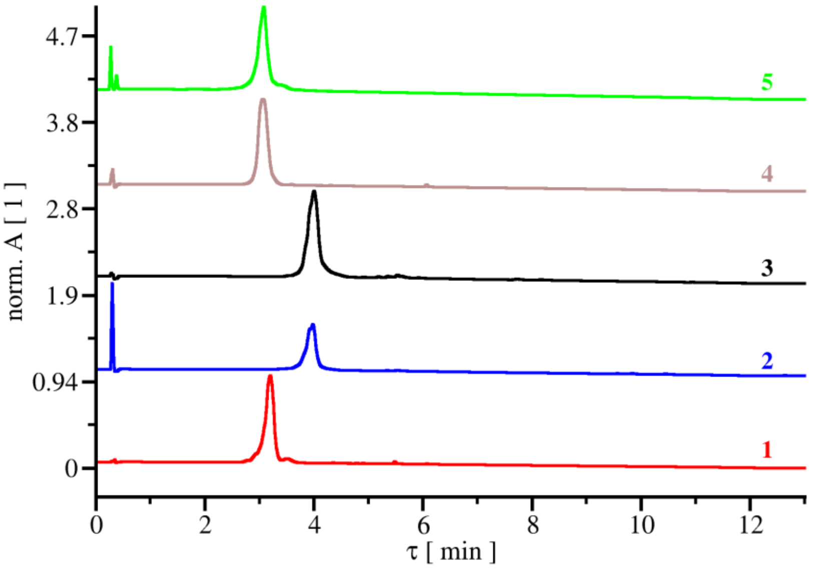

Figure S1. HPLC chromatograms of five prepared peptides using 220 nm detection. **1**: $(PHG)_9PHA$; **2**: $(PPG)_9PPA$**;** **3**: D-$(PPG)_{10}$; **4**: $(HPG)_9HPA$; **5**: $(GHP)_{10}A$

MALDI MS spectra of prepared peptides:

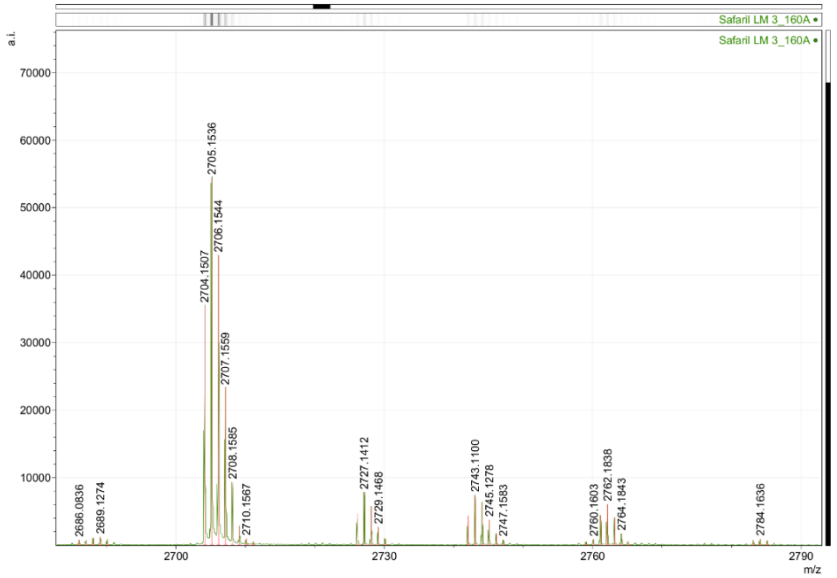

Figure S2. MALDI spectrum of peptide $(PHG)_9PHA$

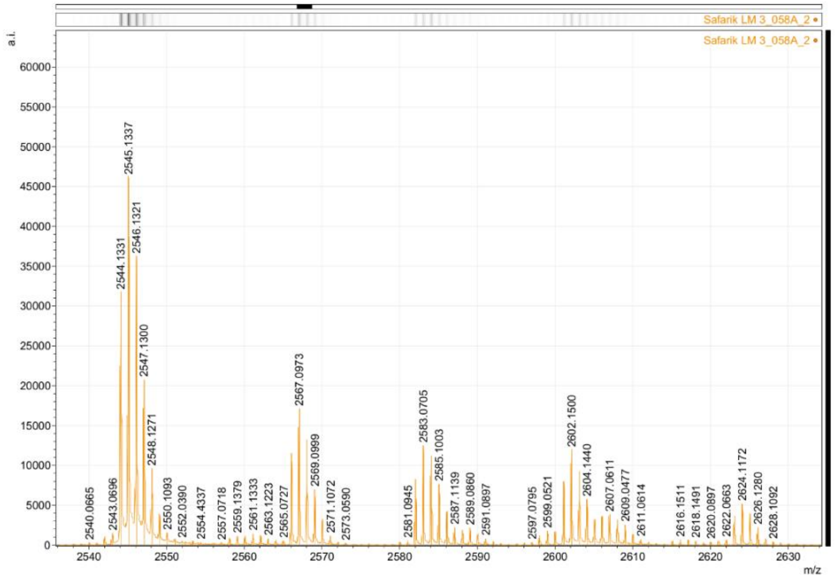

Figure S3. MALDI spectrum of peptide $(PPG)_9PPA$

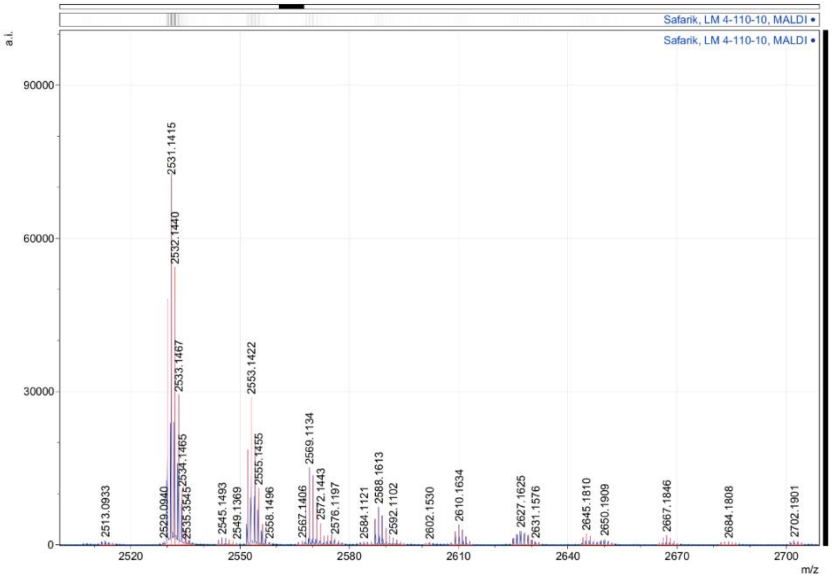

Figure S4. MALDI spectrum of peptide D-$(PPG)_{10}$

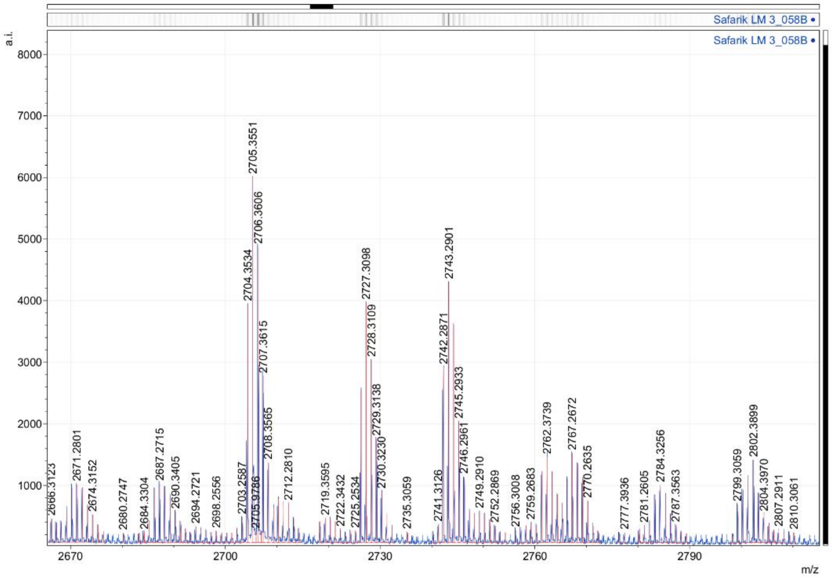

Figure S5. MALDI spectrum of peptide $(HPG)_9HPA$

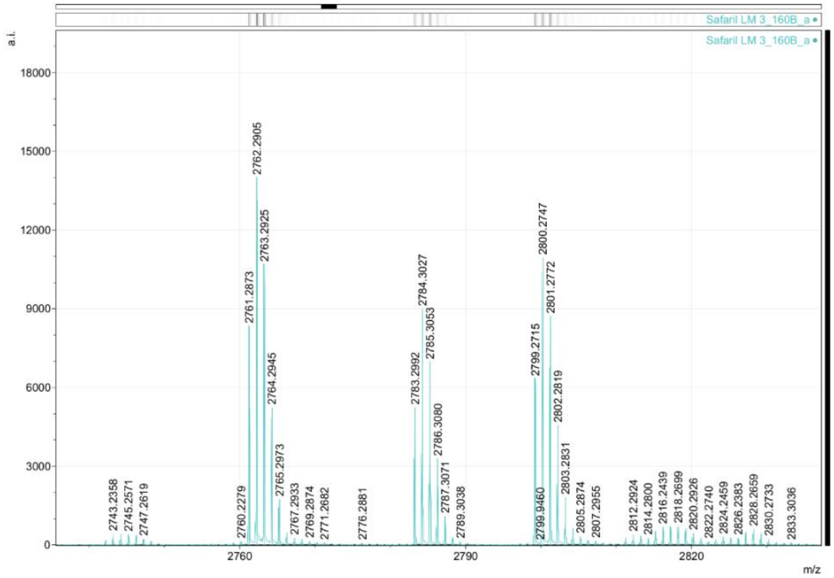

Figure S6. MALDI spectrum of peptide $(GHP)_{10}A$

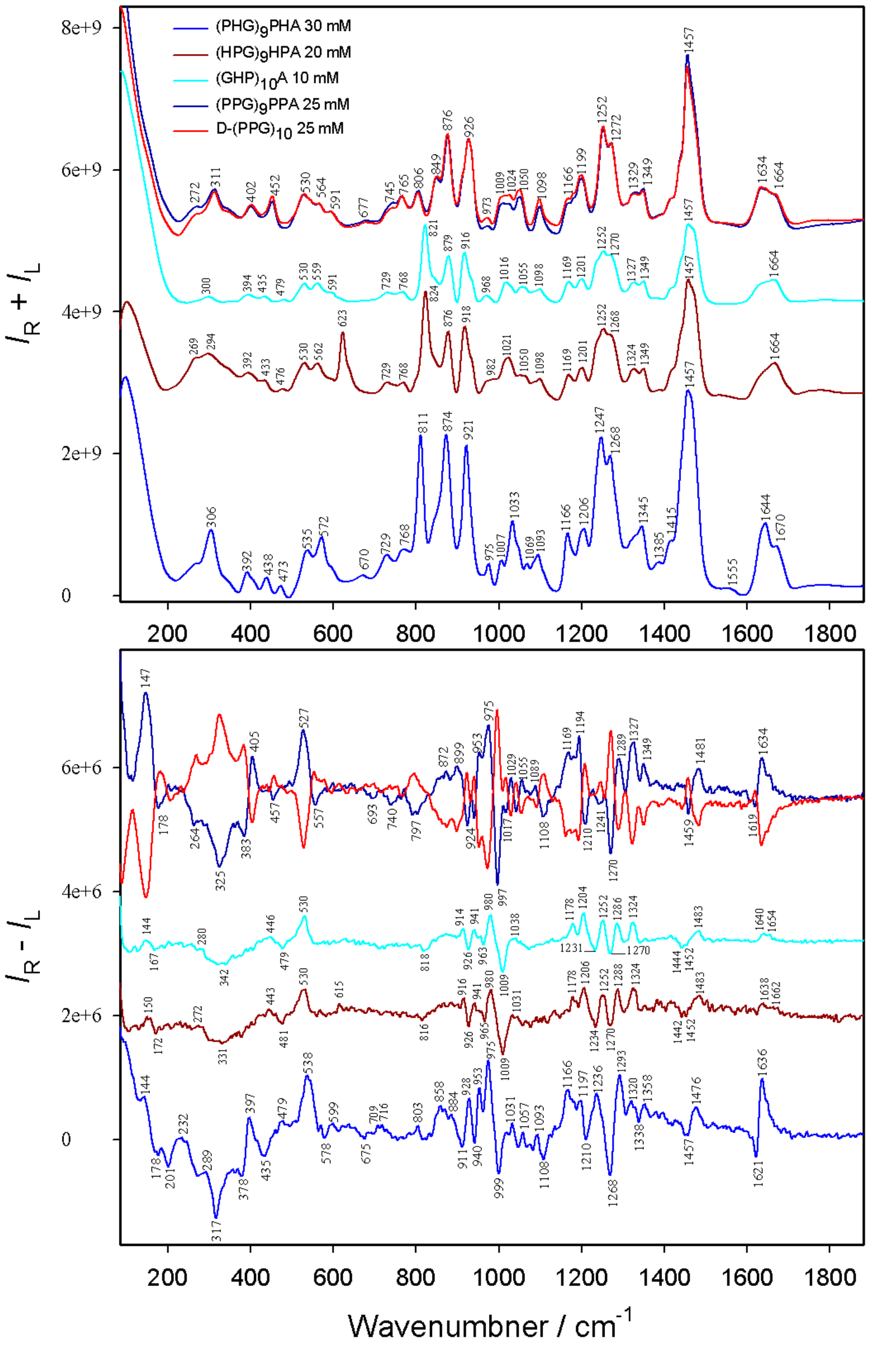

Figure S7. Raman ($I_R + I_L$) and ROA ($I_R - I_L$) spectra of the five collagen-type peptides measured in 0.1 M acetic acid

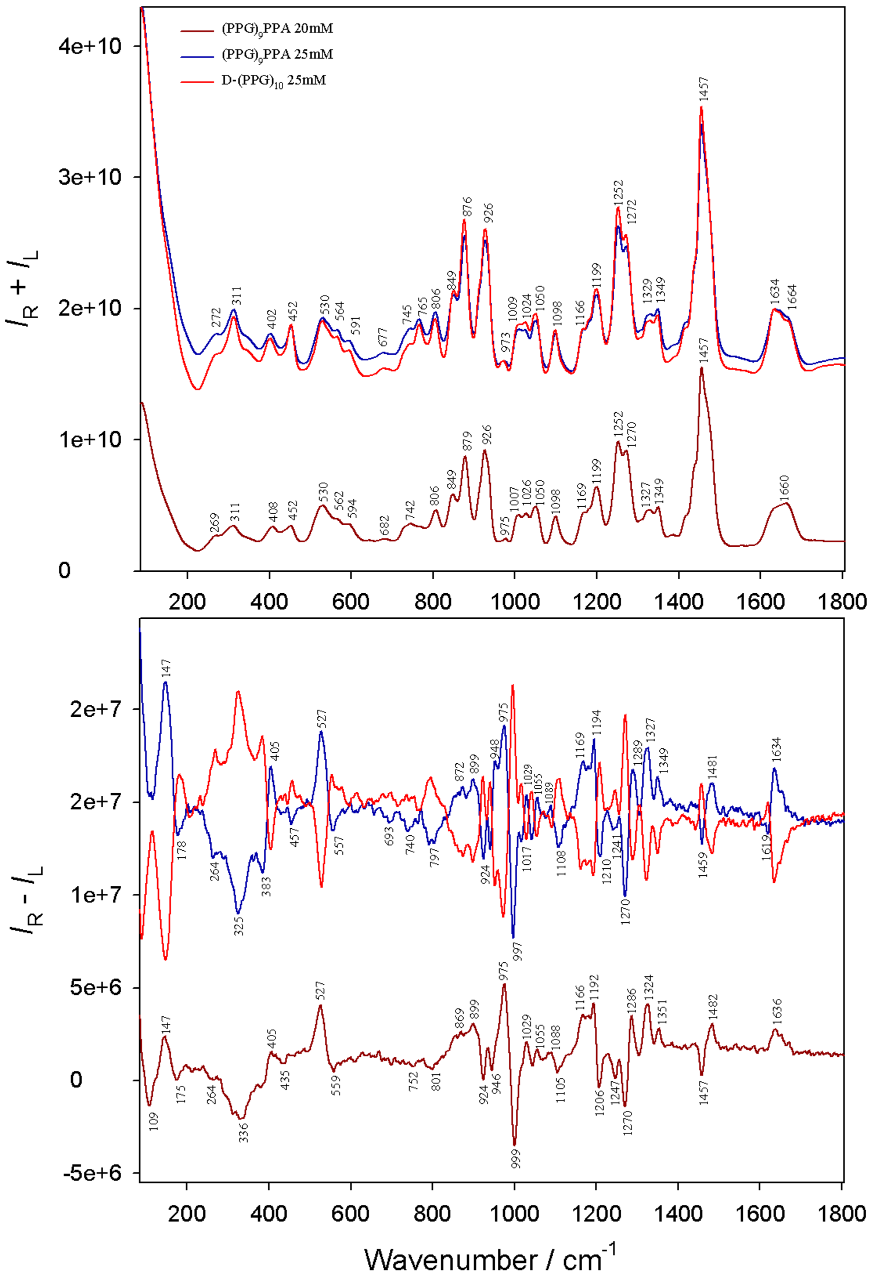

Figure S8. Raman ($I_R + I_L$) and ROA ($I_R - I_L$) spectra of collagen-type peptide (PPG)$_9$PPA at two different concentrations, recorded in 0.1 M acetic acid. The spectrum of D-(PPG)$_{10}$ is included for comparison

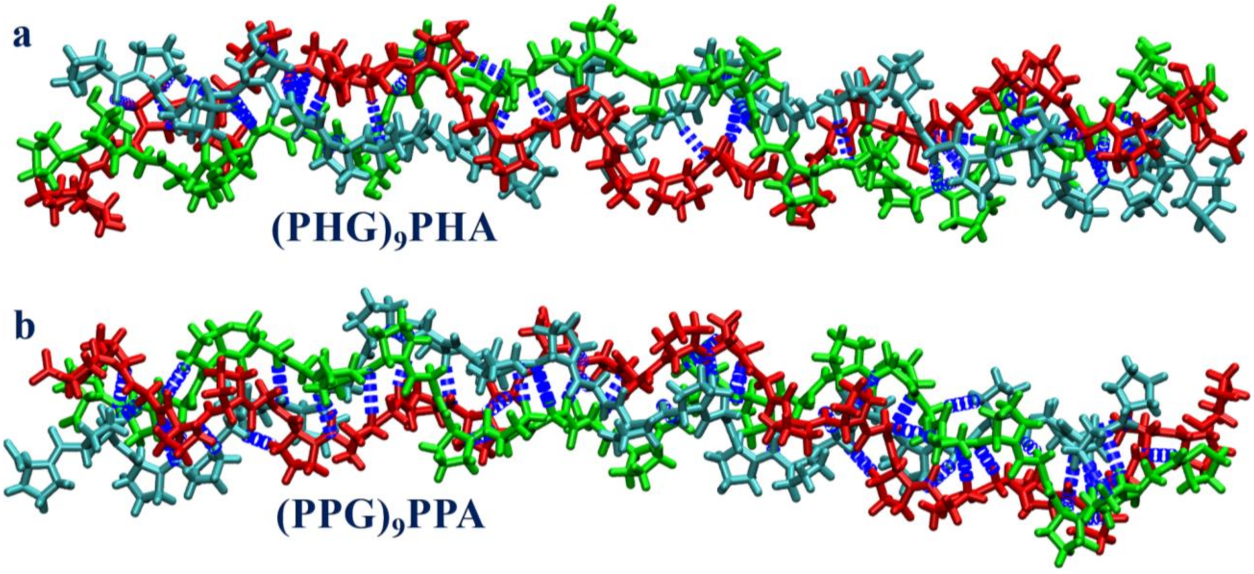

Figure S9. Molecular dynamics (MD) snapshot geometries of $(PHG)_9PHA$ (a) and $(PPG)_9PPA$ (b). Each of the three-polypeptide chains is highlighted in a different colour, and hydrogen bonding between the C=O and N-H groups is indicated by dark blue

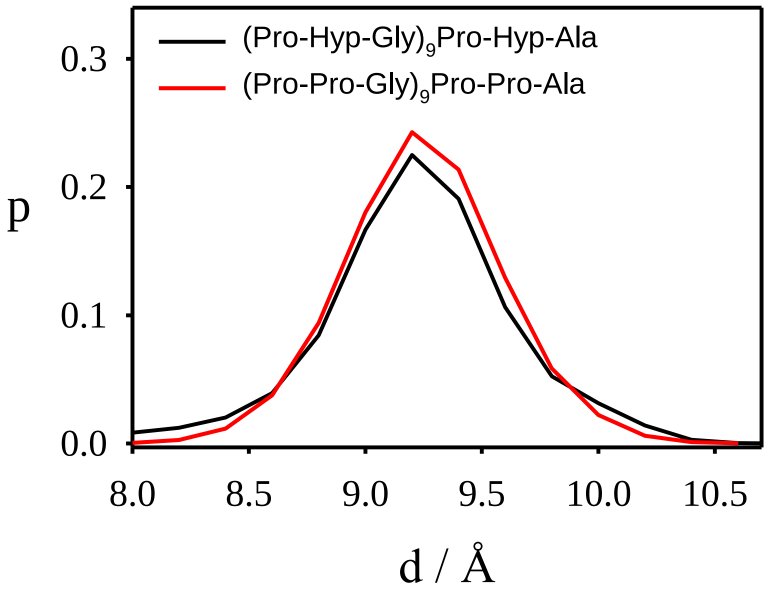

Figure S10. Probability (p) distribution of the tripeptide length defined as the distance between its termini, as obtained by MD simulation. Two segments were analysed, Pro-Hyp-Gly in $(PHG)_9PHA$ and Pro-Pro-Gly in $(PPG)_9PPA$

Table S1. Summary of major Raman bands for collagen proteins and peptides recorded in 0.1 M acetic acid

Wavenumber / cm^{-1}							Band assignment
Proteins		Peptides					
Type I	Type II[c]	$(PHG)_9$ PHA	$(PPG)_9$ PPA	D-$(PPG)_{10}$	$(HPG)_9$ HPA	$(GHP)_{10}A$	
1668[a]<br>1640/1666[b]	1668<br>1640	1670<br>1644	1664<br>1634	1664<br>1634	1664	1664	Amide I<br>ν(C=O)
1455 [a,b]	1455	1457	1457	1455	1457	1457	δCH_3, δCH_2
1345[a,b]<br>1320[a,b]	1345<br>1320	1345	1349<br>1329	1347<br>1329	1349<br>1324	1349<br>1327	C_α-H, CH_2 wagging
1268[a]; 1270[b]<br>1247[a,b]	1270<br>1247	1268<br>1247	1272<br>1252	1270<br>1252	1268<br>1252	1270<br>1252	Amide III<br>δN-H
1206[a,b]	1204	1206	1199	1199	1201	1201	ν(C-C) Hyp/Pro; CH_2 twisting
1178[a,b]	1178						Tyr
1100[a,b]	1100	1093	1098	1098	1098	1098	ν(C-N)
1033[a,b]	1033						Phe; Hyp/Pro ring
		1033<br>1007	1024<br>1009	1024<br>1012	1021	1016	Hyp/Pro ring
1004[a,b]	1004						ν_s(C-C), Phe
		975	973	970	982	968	Hyp/Pro ring deformation
936[b]	938						ν(C-C) of protein backbone
926[a,b]	924	921	926	926	918	916	ν(C-C) of Pro ring
889[b]<br>874[a]	889<br>879	874	876	876	876	879	ν(C-C) of Hyp/Pro ring
856[a,b]	856						ν(C-C) of Pro ring, Tyrosine
			849	849			ν(C-C) of Pro ring
816[a,b]	813						ν(C-C) of protein backbone; ν(C-O-C)
765[a,b]<br>727[a,b]	765<br>722	768<br>729	765<br>745	765<br>742	768<br>729	768<br>729	Amide IV

570	567	572<br>535	591<br>564<br>530	594<br>564<br>530	562<br>530	591<br>559<br>530	Hyp/Pro ring deformation
535	535						S-S vibrations<br>Hyp/Pro ring deformation
473	468	473			476	479	Hyp ring deformation
			452	452			Pro ring deformation
402	402	392	402	402	392	394	Out of plane NH; skeletal deformation
300	306	306	311	311	294	300	Delocalized chain deformation

[a] 23mg/ml

[b] 13 mg/ml

[c] 25 mg/ml

Table S2. Summary of major ROA bands for collagen proteins and peptides recorded in 0.1 M acetic acid

Wavenumber / cm^{-1}							Band assignment
Proteins		Peptides					
Type I[a]	Type II[b]	(PHG)$_9$PHA	(PPG)$_9$PPA	D-(PPG)$_{10}$	(HPG)$_9$HPA	(GHP)$_{10}$A	
1664 (+)<br>1630 (-)	1666 (+)<br>1628 (-)	1636 (+)<br>1621 (-)	1634 (+)<br>1619 (-)	1634 (-)<br>1619 (+)	1662 (+)<br>1638 (+)	1654 (+)<br>1640 (+)	Amide I<br>ν(C=O)
1472 (+)<br>1452 (-)	1465 (+)<br>1439 (-)	1476 (+)<br>1457 (-)	1481 (+)<br>1459 (-)	1483 (-)<br>1457 (+)	1483 (+)<br>1452 (-)<br>1442 (-)	1483 (+)<br>1452 (-)<br>1444 (-)	δCH_3, δCH_2
1340 (-)<br>1315 (+)	1340 (-)<br>1318 (+)	1338 (-)<br>1320 (+)	1349 (+)<br>1327 (+)	1349 (-)<br>1322 (-)	1324 (+)	1324(+)	$C_α$-H, CH_2 wagging
1291 (+)<br>1263 (-)	1286 (+)<br>1266 (-)	1293 (+)<br>1268 (-)	1289 (+)<br>1270 (-)	1288 (-)<br>1270 (+)	1288 (+)<br>1270 (-)	1286 (+)<br>1270 (-)	Amide III, δN-H
1021 (+)<br>999 (-)	1031 (+)<br>1012 (+)<br>999 (-)						Phe; Hyp/Pro ring
		1031 (+)<br>1016 (+)<br>999 (-)	1029 (+)<br>1017 (-)<br>997 (-)	1029 (-)<br>1016 (+)<br>997 (+)	1031 (+)<br>1009 (-)	1038 (+)<br>1009 (-)	Hyp/Pro ring
973 (+)	970 (+)	975 (+)	975 (+)	973 (-)	980 (+)	980 (+)	Hyp/Pro ring deformation
864 (+)	864 (+)	858 (+)	872 (+)	874 (-)			ν(C-C) of Hyp/Pro ring
775 (-)	780 (-)		797 (-)<br>740 (-)	796 (+)<br>755 (+)			Amide IV
556 (+)<br>538 (+)	562 (+)<br>538 (+)	538 (+)	557 (-)<br>527 (+)	554 (+)<br>530 (-)	530 (+)	530 (+)	Hyp/Pro ring deformation
473 (+)		479 (+)			481 (-)	479 (-)	Hyp ring deformation
			457 (-)	457 (+)			Pro ring deformation
325 (-)	331 (-)	317 (-)	325 (-)	325 (+)	331 (-)	342 (-)	Delocalized chain deformation

[a] 23 mg/ml

[b] 25 mg/ml

Table S3. ROA couplet attributed to amide I's C=O stretching in type I and II collagen proteins, and $(PHG)_9PHA$, $(PPG)_9PPA$, and D-$(PPG)_{10}$ peptides

	CID_1 / ν (cm^{-1})	CID_2 / ν (cm^{-1})	$A_{CID} = CID_1 – CID_2$
type I [a]	4.28×10^{-4}/1664	-4.30×10^{-4}/1628	8.58×10^{-4}
type I [b]	1.64×10^{-4}/1664	-4.72×10^{-4}/1630	6.36×10^{-4}
type I [c]	9.17×10^{-5}/1672	-2.58×10^{-4}/1628	3.50×10^{-4}
type II [d]	6.19×10^{-4}/1666	-1.70×10^{-4}/1628	7.89×10^{-4}
$(PHG)_9PHA$	8.95×10^{-4}/1636	-7.81×10^{-4}/1621	1.68×10^{-3}
$(PPG)_9PPA$	6.14×10^{-4}/1634	-2.57×10^{-4}/1619	8.71×10^{-4}
D-$(PPG)_{10}$	-5.22×10^{-4}/1634	2.96×10^{-4}/1619	-8.18×10^{-4}

[a] 13 mg/ml

[b] 23 mg/ml

[c] 50 mg/ml; desaturated due to the temperature (50 °C) required to dissolve the sample

[d] 25 mg/ml
